# A Physiological Overview of the Demands, Characteristics, and Adaptations of Highly Trained Artistic Swimmers: a Literature Review

**DOI:** 10.1186/s40798-019-0190-3

**Published:** 2019-05-14

**Authors:** Eric Viana, David J. Bentley, Heather M. Logan-Sprenger

**Affiliations:** 1 University of Ontario Institute of Technology, 2000 Simcoe Street North, Oshawa, Ontario Canada; 2 Canadian Sport Institute Ontario, 857 Morningside Avenue, Toronto, Ontario Canada

**Keywords:** Physiology, Artistic swimming, Aerobic, Anaerobic, Characteristics

## Abstract

Artistic swimming (AS) is a very unique sport consisting of difficult artistically choreographed routines ranging in the number of athletes (one to ten: solo, duet, team, combination, highlight routine) and with elements performed quickly and precisely above, below, and on the surface of the water. As a result, the physical and physiological demands placed on an athlete are unique to the sport with the most pronounced adaptation being the bradycardic response to long apneic periods spent underwater while performing strenuous movements. This indeed influences training prescription and the desired training outcomes. This review paper explores the physiological demands of AS, the physiological characteristics that influence AS performance, and innovative approaches to enhancing training and performance in elite performers.

## Key Points

- Artistic swimming is a sport that has been largely untouched by the scientific community, allowing subsequent, novel research to be conducted on this unique population.
- As a result of the unique demands of the sport and high training volume, artistic swimming athletes have acquired some unique physiological adaptations, such as the bradycardic response and a blunted hypoxic ventilatory response.

## Background

Artistic swimming (AS), formerly known as synchronized swimming, is an esthetic sport that is judged on technical skill, body positioning, synchronicity, and artistic ability of choreographed movements in water to music [1]. AS comprises *free* as well as *technical* events (routines) and has changed over time since its Olympic debut in 1984 [2]. In the latest revision of Fédération internationale de natation (FINA) regulations, the time limit on the *free* combination routine was reduced from 4:30 to 4:00 [3]. An AS routine is defined as a collection of *elements* artistically strung together and choreographed to music and may utilize costume themes. Over time, AS has become increasingly demanding, incorporating more elaborate lifts, jumps, and aerobatic skills [4, 5]. FINA, the sport’s governing body, alters the technical requirements and mandatory *elements* of routines every 4 years [6]. Elements, also referred to as figures, are compulsory body positions and motions set by FINA. AS routines vary in duration, the number of athletes performing the routine (solo, duet, and multiple team events), and the technical requirements of the routine. Routines can be classified as free or technical with free events lacking compulsory elements and technical routines having compulsory elements [3]. During both technical and free routines, athletes are assessed by three panels of judges, each consisting of five judges, with each panel being responsible for grading execution, impression, and elements [3]. In a free routine, 30% of the final score is based on the execution of all movements, 40% of the final score is devoted to artistic impression, and the final 30% evaluating the difficulty of the movements in the routine [3]. During a technical routine, 30% of the final score is derived from each execution and impression, with the remaining 40% of the score being obtained through the execution and synchronization of elements [3]. When comparing the scoring between free and technical routines, an additional 10% of the final score is allocated to artistic impression during free routines. As the sport of AS has changed, from the requirements and demands of modern routines, the scoring methods have been updated to ensure a greater degree of objectivity in this esthetic sport [7]. These changed include increasing the number of judges from ten (10) to fifteen (15) during the 2013–2017 rule set, with Cronbach *α* values of 0.856 and 0.831 for test-retest internal consistency [7].

The technical routines need to contain mandatory elements that must be performed in order for the athlete(s) to post a valid score [3]. AS athletes may compete in solo, duet, mixed duet, team, free combination, and highlight routines, with the free combination and highlight routines having eight to ten athletes competing simultaneously. Mixed duets have been introduced as an official FINA routine in 2015, where the duet consists of one male and female athlete; however, this review article will focus solely on female artistic swimmers [8]. Hence, solo routines have the potential to elicit different physiological demands relative to duet and team routines.

The purpose of this paper is to review the physical, physiological, and performance characteristics of AS athletes. In addition, the available literature will be used to describe the physiological demands (training and competition) of AS, responses, and adaptations that occur in the athletes participating in artistic swimming. Finally, this review will provide practical recommendations on evidence-based means of enhancing performance in AS.

## Main Text

### Physiological Demands of Artistic Swimming Events

#### Underwater Demands of Artistic Swimming

Artistic swimming places unique demands on the athlete, and perhaps, the most unique to AS is the bradycardic response that is stimulated by the long apneic periods spent underwater (UW) while performing strenuous movements [9]. Indeed, during all AS routines, athletes are required to hold their breath UW during a major portion of a routine while performing vigorous physical activity [9–14]. In previous FINA regulations, select elements, such as the “heron” required the athlete to remain immersed for 45–50 s (s), with a higher score being awarded the more slowly this element is performed [9]: however, anecdotal remarks made by members of Canada Artistic Swimming have suggested that a greater movement frequency improves scoring during international competitions. Some elements require athletes to be inverted during apneic periods, making the breath hold (BH) period more difficult than BH with facial immersion (FI) alone [2].

Davies et al. [9] conducted an analysis of time spent above and under water during free routines and observed BH times ranged from 33 to 66 s, with an average of 43 s. In another study, time-motion analysis was used to record BH times during a Canadian synchronized swimming national championship where the routines of the top 11 Canadian soloists were recorded and subject to time motion analysis [11]. The average BH time was 6.8 s, and any BH shorter than 6.8 s occupied 13% of the total swim routine and 27% of the FI time [15]. As well, Alentejano et al. [11] performed a time-motion analysis of AS during a solo routine and found the longest BH period occurred within the first third of the routine, followed by several short and repetitive BH periods and longer BH towards the end of the routine [11]. Interestingly, the longest BH event was not followed by the longest breathing period [11]. Anecdotal reasoning for the longest BH period at the beginning of the routine may be for the coach/choreographer to ensure a lengthy BH (25.5 ± 6.2 s) is incorporated into the routine, prior the onset of fatigue [10]. However, it is unknown if this trend for the longest BH times in the first third of an AS routine is based purely on artistic choice or if there is some physiological merit to this decision [11]. Some potential physiological reasoning for the long initial BH period may provide a strong stimulus for the bradycardic response, which was followed by several short (< 6.8 s) periods of FI with the athlete able to breathe. These shorter BH periods may not be long enough to stimulate the bradycardic response in AS athletes and more frequent breathing periods could facilitate the exchange of gases in the respiratory system.

It has also been found that solo routines typically have a greater amount of apneic time than duets, team combination, and highlight routines [11], with BH times lasting ~ 40s in length [12, 16]; however, these findings were based on outdated FINA regulations that have changed since these data were published. Free routines may last as long as 4 min (min) with the longest BH time lasting 30s. The team routine is performed with 8 athletes and is ~ 4–5 min with ~ 50% of the routine spent UW [17]. While the variation of physiological responses and effort perception of individual athletes during a team event has yet to clearly demonstrated, some athletes will spend a greater amount of time UW than their teammates. Some athletes may experience UW times closer to 60 s, to support their teammates so they can perform movements above the surface of the water, whose UW times may be closer to 30 s [17]. Additionally, combination and highlight routines are performed with ten athletes, and similar to the team routine, there is a greater variance in UW time when compared to solos and duets. This is especially true when comparing the elements and aerobatic maneuvers seen in current AS routines to those during the sport’s Olympic debut in 1984 [1]. The use of aerobatic maneuvers may alter the athletic and technical skill requirements of the sport and influence the specificity of training prescription. As well, aerobatic maneuvers will increase the UW time of athletes who are positioned at the bottom of a lift and are required to launch a teammate into the air with increased explosiveness [5]. Therefore, during team routines, the roles of team members may differ, influencing the demands of individual athletes within each event. Currently, little is known about individual responses of athletes in team performances. Further research is required to investigate the physiological responses in team compared to solo and duet routines.

#### Physiological Consequences of Breath Hold

The BH and UW effect of AS has a number of implications not least reducing gas exchange and increasing the physiological stress of the sport [13, 18, 19]. The combination of UW exposure and intense rapid muscle contractions creates a physiological environment where gas exchange is limited for the athletes as the vast majority of the energy required during apneic periods must be produced with carbon dioxide accumulation and reduced oxygen availability [13, 19]. Under normal physiologic conditions, respiration is governed by chemoreceptors that are sensitive to the rise in CO_2_ [20]. Therefore, the urge to breathe is governed by the increase in CO_2_, rather than the decrease in O_2_. It has been suggested that the reduction in pH is due to the accumulation of CO_2_ as it cannot be expelled during BH and will accumulate within the blood and muscle tissue [21–23]. In addition to altered physical sensations resulting from long and repeated BH bouts, the accumulation of CO_2_ can impair performance by way of altered cognitive function and in-turn decision-making [24, 25]. This results in a slight increase in the partial pressure of carbon dioxide (pCO_2_) and increase in the partial pressure of oxygen (pO_2_), which can lead to “a dulling of consciousness” and memory impairment [24, 26]. The dulled consciousness and memory impairment may increase the number of errors made during the routine, such as drifting out of position or losing synchronization with the music and teammates, particularly during team and combination routines where there are multiple athletes in the water. Indeed, the number and length of UW exposures has been shown to influence perceived difficulty in AS [19].

#### Pulmonary and Autonomic Physiological Adaptations in Artistic Swimmers

Artistic swimmers are frequently exposed to repeated bouts of UW swimming during training and competition. AS athletes appear to have developed unique physiological characteristics as a result of repeated apneic exposure during training [12, 19, 21, 22, 27]. One possible mechanism, the diving response, decreases cardiac output and causes peripheral vasoconstriction to prioritize the brain and heart with oxygenated blood [26, 28, 29].

When comparing AS athletes to age-matched controls Alentejano et al. [15] demonstrated that AS athletes were able to maintain a BH for a longer period of time compared to their counterparts (110 ± 39 vs. 78 ± 25 s). The AS athletes also showed a greater bradycardic response despite similar end BH pCO_2_. While these experiments were conducted at rest, another study by the same author [15] showed that during upper body exercise (arm cranking), AS athletes demonstrated a greater diving reflex response with FI for 20–25 s compared to a control group. AS athletes were also able to recover better from BH than controls through a more rapid decline in heart rate (HR) and minute ventilation(V_E_) despite having a greater reduction in _PET_O_2_ and greater increase in _PET_CO_2_ [15]. Based upon this greater bradycardic response, more rapid ventilatory recovery from BH, and less blood lactate (La) produced during BH, Alentejano et al. [15] theorized AS may be more efficient at aerobic energy production during apnea.

Naranjo et al. [30] also found that AS athletes were able to better tolerate cycle exercise during BH (without FI) compared to a control group. While the difficulty in routine and elements within a routine may influence the cardiovascular response to exercise in water [31], AS athletes demonstrate more efficient pulmonary function and a greater bradycardic response to exercise in water compared to untrained controls which suggests elite AS athletes may be more efficient at conserving oxygen under exercise stress. It is currently unknown what training age (the number of years engaging in a specific method of training or sport) is required to observe the bradycardic response in AS athletes or whether elite AS athletes display a greater magnitude of response. Therefore, further studies are required to examine the magnitude of physiological and metabolic responses in simulated AS routines and how these responses link to pulmonary characteristics of the athletes.

To help enhance BH times trained artistic swimmers have developed lung adaptations such as a greater vital capacity, total lung capacity, inspiratory capacity, forced expiratory volume (FEV), and forced expiratory volume in one second (FEV_1_) when compared to controls who were matched for seated height [10, 12]. These adaptations have been thought to allow artistic swimmers to increase their BH time at a lower HR by providing a larger reservoir for pulmonary gas exchange [12, 32]. It has been speculated that these respiratory adaptations have led AS athletes to be more efficient at aerobic energy production during BH [30].

There is some speculation that artistic swimmers have a blunted respiratory chemosensitivity and hypoxic ventilatory response (HVR) [12, 15, 33]. Respiratory chemosensitivity is defined as the ability of the brain to detect changes in CO_2_ and alter physiological systems to regulate its levels within tightly controlled parameters [20, 34]. The HVR is the rise in V_E_ associated with decreased O_2_ availability such as acute altitude exposure [35–37], where the respiratory drive is no longer primarily stimulated by hypercapnia (i.e., increase in CO_2_), but hypoxia (i.e., reduction in O_2_) [37, 38].

The relationship between the blunted chemosensitivity, HVR, repeated apneic exposure, and FI might allow artistic swimmers to withstand larger decreases in end-tidal oxygen partial pressure (_PET_O_2_) without altering levels of consciousness (LOC) [10, 33, 39]. Previous authors [12, 21, 22] have theorized that individuals with a lower ventilatory drive are able to withstand a higher partial pressure of arterial carbon dioxide (PaCO_2_) before the urge to breathe overwhelms the will to hold one’s breath and may self-select to sports where this is a benefit, such as AS [22]. Interestingly, in the study conducted by Alentejano et al. [10], they noted the longest BH did not occur on the first trial and hypothesized that anxiety may decrease with subsequent BH trials [40].

These respiratory adaptations to repeated apnea can allow athletes competing in AS to hold their breath longer and at a lower HR despite experiencing greater reductions in SaO_2_ and similar changes in alveolar gases as controls [10]. The respiratory adaptations in AS athletes when compared to controls has been documented; however, the relationship between cardio-respiratory parameters such as forced vital capacity (FVC), FEV_1_, and performance level in AS together with interventions that could improve these parameters have not been well investigated.

#### Circulatory Responses

It has been proposed that splenic contractions may prolong subsequent apneic periods by increasing dissolved gas storage through the release of hematocrit (Hct) after the first BH [41] and prolong future BH times [42, 43]. Hct is the volume of red blood cells (RBC) to the total blood volume of an individual. In mammals, the spleen can serve as a reservoir for RBC, which can be introduced into the circulatory system during exercise and diving [44–46]. The increase in circulating RBC increase the total Hct, which may improve the oxygen-carrying capacity of a given volume of blood and prolong BH times in humans [41].

#### Metabolic Responses to Artistic Swimming and Competition

Research has demonstrated that artistic swimmers are exposed to considerable metabolic demand because of the combination of BH and vigorous exercise [9]. Results from Rodríguez-Zamora et al. [13] indicated moderate to high La_peak_ in junior and senior age categories, ranging from ∼ 5 to 13 mmol·L^−1^, with an overall average of 7.3 mmol·L^−1^ as the mean across all routines. This possibly indicates a considerable anaerobic contribution to the sport. Unfortunately, La_peak_ values from competition are limited with reports on lactate responses during training being more extensive [17, 21, 23, 31, 47]. During training, La values have been documented for individual elements [16, 18, 31, 48], whole routines [17, 18, 47], and other swim tests such as 400-m freestyle [17, 21]. However, extrapolating the La values for individual elements to whole routines is difficult due to most studies not defining what elements were used in each routine. Additionally, the La values for individual elements were obtained under previous technical regulations, and these elements may not be used as frequently since the September 2017 revision [3]. Interestingly, Jamnik et al. [49] reported a La_mean_ of 12.7 ± 1.3 mmol·L^−1^ in five elite artistic swimmers during competition, surprisingly higher than the 7.0 ± 1.3 mmol·L^−1^ when performing the same routine during practice. This finding might indicate that high level AS performers can better tolerate increases in metabolic acidosis or represent a greater glycolytic demand with the reasoning largely unknown. This discrepancy between La_mean_ during practice and competition may be in part due to a period of greater anticipatory pre-activation during competition when compared to practicing the same routines. This has also been theorized by Rodríguez-Zamora et al. [13] to describe the physiological reasoning behind a brief period of tachycardia prior to starting a routine during competition. Additionally, this anticipatory pre-activation may be used to maximize aerobic and anaerobic metabolic stores since apneic diving capacity is determined by asphyxiation tolerance, which is dependant on how rapidly these stores are exhausted during the routine [14, 50]. The available literature has estimated the anaerobic contributions to AS through excess post-exercise oxygen consumption (EPOC) during the first 3 min of recovery, as well as La measurements [21]. Bante et al. [21] speculated the EPOC was used for phosphocreatine resysnthesis, since bursts of anaerobic power are more common during an AS routine rather than a single effort [6]. However, the prolonged and repeated apneic exposures in AS may increase the anaerobic contributions more than other aquatic sports [13, 14]. Though quantifying the anaerobic contributions to an AS routine is difficult, the anaerobic contributions are estimated to be less than that of a 400-m freestyle swim [21], but less than a 200 m freestyle swim [51–53]. Based upon this information, and the work put forth by Rodríguez and Mader [54], one could estimate 40% of the energy demands of an AS routine may be produced anaerobically. However, no literature has quantified the anaerobic contributions of an AS routine which means sport scientists can only speculate on or estimate these anaerobic contributions based on freestyle swimming.

Homma [16] reported that the time spent UW in international competitions was highest in solo (62.2%), followed by duets (56.1%), and then teams (51.2%). It has therefore been speculated that the greater the reduction in peripheral O_2_ delivery, due to the longer or more frequent BH times, the higher the La production due to hypoxemia [12, 16]. This is in line with the results from Rodríguez-Zamora et al. [13] who demonstrated that the highest La_peak_ values were obtained in free solo and duet programs. The authors suggest that the La_peak_ values can be analyzed in terms of the specific influence of the BH periods, the activation of the glycolytic metabolism in the exercising muscles, and the specific training adaptations of the athlete [13]. It has been suggested that the peripheral vasoconstriction associated with the diving response during the BH periods would reduce the blood supply to the muscles and lower their O_2_ stores. As a result, if the energy turnover in the exercising muscles is sustained or increased, a greater proportion of energy will be derived via glycolytic metabolism and result in greater La production [28, 55, 56].

The higher La_peak_ values obtained in solo and duet competitive routines (∼ 3–3.5 min) suggest a more intense activation of anaerobic glycolysis [23]. It has been documented that free programs usually start with an UW sequence, with highly placed contestants incorporating longer BH times into their routines, with some BH times in excess of 45 s [9]. In light of the diver’s response and subsequent peripheral vasoconstriction and redistribution, oxygen stores may be reduced at the onset of the routine causing the working muscles to receive less oxygen than required resulting in the muscle-derived energy from glycolytic metabolism [13, 16]. Additionally, authors have suggested that the difficulty and order of the figures could influence the course of activation of glycolysis in the exercising muscles [13, 16]. For instance, the rate of execution of skill elements has a tendency to be higher in the solo (50%) than in duet and team (32%) events [8, 16]. As such, the solo is composed of more figure parts implying a higher physiological stress, potentially demanding a greater reliance on glycolytic metabolism contributing to the higher La during competition [13]. Moreover, pool-based and dryland training to enhance the athlete’s lactate handling and profile should be a focus of training with the intention of preventing premature fatigue during competition. However, it is yet to be determined whether minimizing La appearance through potential ergogenic aids (e.g., sodium bicarbonate) is associated with better performance or reduced perceived effort during competition.

### Physiological Characteristics Influencing Performance of Artistic Swimming

#### Aerobic Capacity

Given the unique constraints on respiratory exchange and metabolic demands in AS, it is pertinent to examine the significance of key physiological and performance in AS athletes. An elevated maximal oxygen uptake (VO_2max_) has been shown to be an important requirement of a number of other sports [57–60]. The majority of studies conducted in AS athletes have examined VO_2max_ in mixed cohorts and have used a variety of exercise challenge tests to induce a maximal response. Roby et al. [61] found a mean VO_2max_ of 43 ml/kg/min when measured in tethered swimming which did not differ from a group of untrained individuals. Therefore, these authors concluded that aerobic capacity was not a factor in AS performance. In another study, Poole et al. [62] ascertained a similar mean VO_2max_ of 44 ml/kg/min during cycling in the Canadian national artistic swimming team. Of interest is the VO_2max_ ascertained correlated with scores during a solo routine (*r* = 0.41, *p* = 0.06) with the authors concluding that aerobic capacity was an important factor in fatigue during AS routine. Yamamura et al. [53] confirmed this finding and found performance scores in a group of well-trained AS correlated with relative VO_2max_ (50.8 ± 2.8 ml/kg/min) when tested in a swimming flume (*r* = 0.71, *p* < 0.05). Other studies have attempted to examine peak VO_2_ during free swimming and compared to that obtained during a simulated event. Bante et al. [21] found VO_2_ was significantly higher after a 400-m swim versus a simulated AS routine, Chatard et al. [17] found that VO_2max_ measured after a 400-m freestyle swim improved with a 5-week period of AS training and the change in VO_2max_ was positively correlated with performance during a synchronized swimming routine. Finally, Sajber et al. [63] used a variation of the land-based multi-stage shuttle test (MSST) in a 25-m pool and found the total duration of the MSST strongly correlated with AS performance score at a national championship (*r* = − 0.81) indicating the longer the swim time, the higher the score. This study also demonstrates that measuring VO_2max_ of AS athletes while swimming might be more appropriate than doing so when running or cycling.

It has also been suggested that AS is a sport that requires both aerobic and anaerobic power [6], largely due to the long apneic periods spent UW while performing strenuous movements [9]. Despite this, there is a scarcity of literature that has examined the anaerobic power or capacity of AS athletes. The lack of literature may be in part due to the absence of a valid sport specific assessment and the difficulty of conducting metabolic measurements in AS [21]. Anaerobic capacity is typically determined by a maximal exercise test with accompanying oxygen costs measured relative to maximal aerobic capacity, such as the Wingate anaerobic test (WANT) [64–68]. Based on these data presented by Jamnik et al. [49], the anaerobic power produced during the WANT (6.0 ± 0.2 watts/kg) ranked the participants poorly when compared to active young adults, falling between the 10th and 20th percentile for females [69]. In order for this approach to be valid, an in-water test specific to the demands of AS should be conducted, despite the WANT being the gold standard field test for measuring anaerobic power [70]. The lack of significant correlation between anaerobic capacity and performance score may be due to the low specificity of conventional anaerobic tests, like WANT, where the anaerobic tests require a sustained high-intensity effort. Unlike in AS, there are shorter, high-intensity efforts interspersed with lower intensity periods where the athletes have the opportunity to recover [53]. As in other aquatic sports, collecting oxygen cost data is challenging. In AS, anaerobic fitness may prove to be an important measure due to the prolonged and repeated bouts of BH with FI. In summary, the relative importance and aerobic and anaerobic fitness in AS athletes is not clear because of the means of assessment and cohort that has been tested. There is further work required to establish whether aerobic fitness is important in elite AS athletes and how this variable is related to response to simulated routines.

### Innovative Approaches to Improving Performance in Artistic Swimming

The sport of AS requires a significant contribution from both aerobic and anaerobic metabolism with the contribution of each energy system influenced by prolonged periods UW [13, 19]. Combined with the metabolic demands of the sport, athletes are required to learn and deliver highly choreographed and technical movements under extreme physiological stress. Therefore, innovative approaches to training and competition represent key areas for those working in this sport. While the training specifics required in AS competitors are not well understood, a significant total volume of training has been demonstrated in elite AS athletes [71, 72]. Indeed, the training approaches in AS is not well understood with quantification of training load difficult due to the UW nature of training and competition. Therefore, the optimal training approach for general and sport-specific performance improvements in AS has not been defined. In terms of sport specificity, it makes sense that AS should practice and rehearse the technical requirements of the event but also utilize complementary training approaches in order to target the specific demands of the sport. For instance, due to the UW nature AS competitions, practicing prolonged periods UW combined with intense muscle contraction could be utilized in combination with technical elements to improve overall AS performance. Previous studies in swimming has suggested short term periods of swimming with controlled/regulated breathing frequency or full apnea results in an elevated pulmonary function and capacity [73–75], which in turn may improve oxygen demand during periods UW through repeated periods of hypercapnia and the associated increase in _P_CO_2_ and decrease in pH, all of which serve as mechanisms to encourage physiological adaptation [76–78]. In addition, other studies advocate the use of respiratory muscle training to improve pulmonary function and improve swimming performance [79]. BH training could also be used in relatively young new athletes, or athletes whose bradycardic response is not as pronounced. This could increase BH duration by reducing the anxiety associated with prolonged BH times seen during AS routines [10, 40, 41]. Improving maximal BH could allow greater artistic expression when the athlete, coach, and choreographer are designing a routine ahead of the competition. Additionally, BH training could enhance breath control, allowing athletes to perform elements that require lengthy breath holds, such as the heron, with less difficulty [12, 22]. However, it is currently unknown whether this type of training has the potential to improve performance in AS, as the efficacy of respiratory muscle and BH training have not been investigated in AS. When examining the literature of competitive swimming, improvements in physiological characteristics such as VO_2peak_, La clearance, and improvement in energy efficiency while performing the same workload are thought to be important for performance in other aquatic sports, such as AS [23, 80]. Anecdotal reports indicate that elite AS athletes are able to perform pure swimming (front crawl and form strokes) exceptionally well, and performances in swimming are recognized as a key element of AS preparation. Indeed, after a 5-week training intervention, there was a decrease in VO_2_ and La during a 400-m freestyle swim [17], which suggests the athletes became more efficient at aerobic energy production and La handling which could indirectly influence the capacity to perform AS events.

Improvement of sculling and the egg beater kick either with specific water training or dryland training may be of benefit to AS athletes from an injury prevention and locomotion perspective. Sculling and the eggbeater kick are two main methods of movement during AS routines. Sculling is a series of repeated arm movements that can be used for stabilization, locomotion, and altering the body’s position such as entering or exiting inversion [81, 82]. The eggbeater kick can occupy as much as 40% of an AS routine and is especially useful in a team and combination routine because there are multiple athletes performing different roles [81]. Ultimately, improving localized fatigue resistance in these movements might result in improved overall efficiency and decrease the demand of the event which in turn may lead to better performance.

The metabolic response during AS events indicates a significant acidic environment which could influence performance during AS competitions [13, 49]. Ergogenic aids such as beta-alanine and sodium bicarbonate may improve performance in other anaerobically orientated and non-esthetic sports, such as water polo [83, 84], which could be introduced to AS athletes and coaches to improve the performance during competition [85–88]. However, the effects of these supplements on the physiological responses and performance in AS events have not yet been investigated.

Finally, it is well known that actual altitude training evokes changes in circulatory markers and endurance performance in other sports [89, 90]. Typically, prolonged periods of living and/or training at moderate altitude [live high-train low (LHTL) or live high-train high (LHTH)] evoke increases in red cell mass which has been linked to concurrent increases in aerobic capacity [91, 92]. Interestingly, improvements in hemoglobin mass in water polo players after 13 days of LHTL [93], which may provide a stimulus to increase VO_2peak_ in AS athletes, have been positively correlated with performance [62]. Recently, anaerobic/sprint training in simulated hypoxia has been reported as an alternative hypoxia training approach resulting in improved repeated sprint or anaerobic performance in trained athletes [94, 95]. It seems apparent that such adaptations to simulated or actual hypoxia training could be applied to AS either by way of sport-specific training at moderate altitude or generalized anaerobic training in hypoxia. Future studies are required to examine the efficiency of such approaches for AS performance.

## Summary and Conclusion

AS is a physiologically unique and demanding sport that elicits specific training adaptations as a result of chronic exposure to BH and FI. Research is consistent in demonstrating the novel adaptations to AS training (i.e., bradycardic response); however, little is known about the time course in acquiring these adaptations with AS training. Although there is some research regarding the specific elements of AS, there are few data characterizing the physical and physiological correlates to AS performance and the impact of scored performance and the relationship to physiology. Ultimately, elucidating this information will improve the specificity of training prescription to optimize AS specific physiology, while allowing more time spent on choreography and technical skill. Furthermore, innovative strategies to improve performance are suggested, and while needing to be further explored, these include extending BH duration through respiratory training, AS-specific swim training, the use of dietary ergogenics, and intermittent hypoxic training.

## Abbreviations

AS: Artistic swimming
BH: Breath hold
CO_2_: Carbon dioxide
EPOC: Excess post-exercise oxygen consumption
FEV: Forced expiratory volume
FEV1: Forced expiratory volume in one second
FI: Facial immersion
FINA: Fédération internationale de natation
FVC: Forced vital capacity
Hct: Hematocrit
HR: Heart rate
HVR: Hypoxic ventilatory response
La: Lactate
La_peak_: Peak lactate
LHTH: Live high-train high
LHTL: Live high-train low
LOC: Levels of consciousness
MSST: Multi-stage shuttle test
O_2_: Oxygen
PaCO_2_: Partial pressure of arterial carbon dioxide
PaO_2_: Partial pressure of arterial oxygen
pCO_2_: Partial pressure of carbon dioxide
_PET_CO_2_: Partial pressure of end tidal carbon dioxide
_PET_O_2_: Partial pressure of end tidal oxygen
pO_2_: Partial pressure of oxygen
RBC: Red blood cells
UW: Underwater
VE: Minute ventilation
VO_2max_: Maximal oxygen uptake
VO_2peak_: Peak oxygen uptake
WANT: Wingate anaerobic test
